# Analysis of CC chemokine and chemokine receptor expression in solid ovarian tumours

**DOI:** 10.1054/bjoc.2001.2020

**Published:** 2001-09

**Authors:** C Scotton, D Milliken, J Wilson, S Raju, F Balkwill

**Affiliations:** ICRF Translational Oncology Laboratory, St Bartholomew's and Royal London School of Medicine and Dentistry, Science Building, Charterhouse Square, London, EC1M 6BQ, UK; Department of Obstetrics and Gynaecology, Guy's and St Thomas' Hospital Trust, Lambeth Palace Road, London, SE1 7EH, UK

**Keywords:** chemokine, ovarian cancer, CCR1, hypoxia

## Abstract

To understand the chemokine network in a tissue, both chemokine and chemokine receptor expression should be studied. Human epithelial ovarian tumours express a range of chemokines but little is known about the expression and localisation of chemokine receptors. With the aim of understanding chemokine action in this cancer, we investigated receptors for CC–chemokines and their ligands in 25 biopsies of human ovarian cancer. CC–chemokine receptor mRNA was generally absent from solid tumours, the exception being CCR1 which was detected in samples from 75% of patients. CCR1 mRNA localised to macrophages and lymphocytes and there was a correlation between numbers of CD8^+^ and CCR1 expressing cells (*P* = 0.031). mRNA for 6 CC-chemokines was expressed in a majority of tumour samples. In a monocytic cell line in vitro, we found that CCR1 mRNA expression was increased 5-fold by hypoxia. We suggest that the CC-chemokine network in ovarian cancer is controlled at the level of CC-chemokine receptors and this may account for the phenotypes of infiltrating cells found in these tumours. The leukocyte infiltrate may contribute to tumour growth and spread by providing growth survival factors and matrix metalloproteases. Thus, CCR1 may be a novel therapeutic target in ovarian cancer. http://www.bjcancer.com © 2001 Cancer Research Campaignhttp://www.bjcancer.com


*British Journal of Cancer* (2001) **85**(6), 891–897
© 2001 Cancer Research Campaign
doi: 10.1054/ bjoc.2001.2020, available online at http://www.idealibrary.com on IDEAL
http://www.bjcancer.com

### Analysis of CC chemokine and chemokine receptor expression in solid ovarian tumours

**C Scotton[1], D Milliken[2], J Wilson[1], S Raju[2] and F Balkwill[1]**

[1]ICRF Translational Oncology Laboratory, St Bartholomew's and Royal London School of Medicine and Dentistry, Science Building, Charterhouse Square, London EC1M 6BQ, UK; [2]Department of Obstetrics and Gynaecology, Guy's and St Thomas' Hospital Trust, Lambeth Palace Road, London SE1 7EH, UK

**Summary** To understand the chemokine network in a tissue, both chemokine and chemokine receptor expression should be studied. Human epithelial ovarian tumours express a range of chemokines but little is known about the expression and localisation of chemokine receptors. With the aim of understanding chemokine action in this cancer, we investigated receptors for CC–chemokines and their ligands in 25 biopsies of human ovarian cancer. CC–chemokine receptor mRNA was generally absent from solid tumours, the exception being CCR1 which was detected in samples from 75% of patients. CCR1 mRNA localised to macrophages and lymphocytes and there was a correlation between numbers of $CD8^+$ and CCR1 expressing cells ($P = 0.031$). mRNA for 6 CC-chemokines was expressed in a majority of tumour samples. In a monocytic cell line in vitro, we found that CCR1 mRNA expression was increased 5-fold by hypoxia. We suggest that the CC-chemokine network in ovarian cancer is controlled at the level of CC-chemokine receptors and this may account for the phenotypes of infiltrating cells found in these tumours. The leukocyte infiltrate may contribute to tumour growth and spread by providing growth survival factors and matrix metalloproteases. Thus, CCR1 may be a novel therapeutic target in ovarian cancer. © 2001 Cancer Research Campaign http://www.bjcancer.com

**Keywords**: chemokine; ovarian cancer; CCR1; hypoxia

Chemokines are chemoattractant cytokines, characterised by the presence of a conserved cysteine motif adjacent to the N-terminus. They are subdivided into 2 major groups: the CC chemokines and the CXC chemokines, in which the first 2 cysteine residues are either adjacent or separated by one amino acid, respectively (Clore and Gronenborn, 1995). Individual chemokines selectively attract leukocyte subsets through chemoattraction and by activating leukocyte integrins to bind their adhesion receptors on endothelial cells (Ebnet and Vestweber, 1999). The CXC chemokines act predominantly on neutrophils and T-lymphocytes, while the CC chemokines are active on various cell types, including monocytes and lymphocytes. Chemokines exert their effects by binding to 7 transmembrane domain G protein-coupled receptors. 11 receptors for the CC chemokines (CCR1-11) and 5 receptors for the CXC chemokines have been identified (Wang et al, 1998; Schweickart et al, 2000; Zlotnik and Yoshie, 2000). Their ligands bind to the extracellular N-terminus, leading to phosphorylation of serine/threonine residues on the cytoplasmic C-terminus, signalling and receptor desensitisation (Turner et al, 1998). There is apparent redundancy in the system; each receptor can respond to more than one chemokine; most chemokines can use more than one receptor, and each leukocyte subset may express several receptors. Yet the importance of individual chemokines is shown in transgenic and knockout mice (Gao et al, 1997; Kurihara et al, 1997; Lu et al, 1998). Specific chemokines are associated with distinct inflammatory infiltrates in a number of diseases, and chemokine antagonists have activity in experimental models (Takeya et al, 1993; Car et al, 1994; Baggiolini and Moser, 1997; Howard et al, 1999). However, in most tissues there is likely to be a complex chemokine/receptor network.

We have been studying the chemokine network in human epithelial ovarian cancer. This tumour microenvironment is a variable mixture of epithelial tumour cells, stromal fibroblasts, endothelial cells and infiltrating leukocytes, which are mainly $CD68^+$ macrophages and $CD8^+$ lymphocytes (Negus et al, 1997). The cytokine context of these tumours generally comprises inflammatory cytokines, growth factors and chemokines, but there is a lack of lymphocyte-associated cytokines (Burke et al, 1996).

The presence of macrophages and lymphocytes in this tumour microenvironment is related to local production of chemokines (Bottazzi et al, 1985; Yoong et al, 1999). We reported a relationship between lymphocyte and macrophage counts in ovarian tumours and mRNA expression of the CC chemokine CCL2 (MCP-1) by tumour cells and macrophages (Negus et al, 1997). The cytokine TNF-$\alpha$, also present in the epithelial tumour islands may regulate this CCL2 production (Negus et al, 1995). We also found that mRNA for other CC chemokines, CCL3 (MIP-1$\alpha$), CCL4 (MIP-1$\beta$) and CCL5 (RANTES), localised to leukocytes in the tumour (Negus et al, 1997), suggesting that a chemokine network existed. However, it was not clear how this network was controlled. Changes in the profile of chemokine receptors expressed by individual cells can inhibit cell migration or change their path. Thus, to understand the chemokine network in a tissue, both chemokine and chemokine receptor expression must be studied.

In this paper we have compared CC chemokine receptor mRNA expression in solid tumours with the expression of CC chemokine mRNA. In spite of abundant CC chemokine expression in ovarian tumours, we have found that CC chemokine receptor expression is weak or absent in the solid tumour microenvironment. CCR1

*Received 19 March 2001*
*Revised 6 June 2001*
*Accepted 11 June 2001*

*Correspondence to:* F Balkwill

(whose ligands include CCL3, CCL4, CCL5 and CCL7 (MCP-3)) was the only chemokine receptor consistently expressed in the solid tumours. This receptor localised to infiltrating $CD68^+$ macrophages and $CD8^+$ lymphocytes. There was no evidence that the CC chemokine receptors studied were expressed by epithelial tumour cells. The microenvironment of solid tumours may down-regulate expression of some chemokine receptors and we present preliminary evidence that hypoxia, which is a common feature of solid tumours (Vaupel et al, 1998), may up-regulate CCR1.

### MATERIALS AND METHODS

### Samples

25 biopsies from human ovarian tumours were obtained at operation and snap frozen into liquid nitrogen. These were classified as serous adenocarcinoma (19), clear cell carcinoma (2), mucinous adenocarcinoma (1), anaplastic carcinoma (1), signet ring carcinoma (1) and endometrioid carcinoma (1). Sections for in situ hybridisation were mounted on baked glass slides coated with 3-aminopropyl-triethoxy-silane, air dried, and stored at –70˚C. Serial sections were cut onto poly-L-lysine-coated slides and stored at –70˚C. Control peripheral blood mononuclear cells (PBMC) were isolated from heparinised venous blood or cytophoresis buffy coats from normal volunteers by Ficoll-Hypaque density centrifugation (Lymphoprep™, Nycomed, Birmingham, UK).

### RNA extraction and RT-PCR

Total RNA was prepared from all samples using Tri Reagent™ (Sigma, Poole, UK). Solid tumour biopsies were homogenised in Tri Reagent™ using an Ultra-turrax T25 tissue homogeniser (Janke & Kunkel, Staufen, Germany). For RT-PCR and RNase protection analysis, total RNA was DNase-treated to remove contaminating genomic DNA, using RNase-free DNase I (Pharmacia Biotech, St Albans, UK). cDNA was synthesised from DNase-treated total RNA using the Ready-to-Go™ T-primed First Strand kit (Pharmacia Biotech, UK). The primers for CCL2, CCL22 (MDC) and CCR1 were designed from sequences submitted to Genbank, using Primer 3.0. CCL3 and CCL4, CCL5 and CCL8 primer sequences were from (Hosaka et al, 1994), (Mattei et al, 1994) and (Van Coillie et al, 1997) respectively. The primer sequences and product sizes for CCL2, CCL22 and CCR1 are:

| | | |
|---|---|---|
| CCL2 | For | CAAACTGAAGCTCGCACTCTCGCC |
| | Rev | ATTCTTGGGTTGTGGAGTGAGTGTTCA |
| | Product = 354 bp | |
| CCL22 | For | CCCTACCTCCCTGCCATTAT |
| | Rev | CAGGGAGCTAGAACCCAACA |
| | Product = 338 bp | |
| CCR1 | For | AAAGCCTACGAGAGTGGAAGC |
| | Rev | AGAGGAAGGGGAGCCATTTA |
| | Product = 426 bp | |

25 μl volume per sample was used, containing 200 ng cDNA, 1 U AmpliTaq DNA polymerase, GeneAmp PCR buffer, GeneAmp dNTPs (all from Perkin Elmer, Beaconsfield, UK) and 4μM each primer. The following protocol was used in a GeneAmp® PCR System 9700 thermal cycler: 94˚C (5 min); 35 cycles 94˚C (30 s), 60˚C (30 s), 72˚C (30 s); 72˚C (7 min). PCR products were electrophoresed through 1.2% agarose gel and visualised by ethidium bromide. 123 bp markers (Gibco BRL, Paisley, UK) were used to estimate band sizes. PCR products were gel extracted and sequenced to confirm their identity.

### RNase protection assay (RPA)

The hCR5 template set from Pharmingen (Becton Dickinson, Oxford, UK) contained DNA templates for CCR1, CCR2, CCR2a, CCR2b, CCR3, CCR4, CCR5, CCR8, GAPDH and L32. RPA was carried out using [$\alpha^{35}$S]UTP (Amersham International plc, Aylesbury, UK) instead of [$\alpha^{32}$P]UTP. The RNase-protected fragments were run out on an acrylamide-urea sequencing gel (BioRad Laboratories Ltd, Hemel Hempstead, UK), which was then adsorbed to filter paper and dried under vacuum. Autoradiography was subsequently carried out using Kodak Biomax MS film with a Transcreen LE-intensifying screen (Sigma).

### In situ hybridisation (ISH)

[$\alpha^{35}$S]UTP-labelled antisense and sense riboprobes were generated from 1100 bp fragments of CCR1 and CCR4 cDNA cloned in pcDNA1 (Stratagene, Cambridge, UK), using Sp6 and T7 RNA polymerases (Promega Ltd, Southampton, UK). These cDNAs were a kind gift from Antonio Sica (Mario Negri Institute, Milan, Italy). Antisense β-actin was used as a positive control in all experiments. In situ hybridisation was carried out using the method described in (Naylor et al, 1990). Image capture was with Image Grabber PCI (Neotech Ltd, London, UK).

### Immunohistochemistry

Cryostat sections were fixed in 4% paraformaldehyde in PBS for 5 minutes. Sections were preincubated with normal rabbit serum (DAKO, Ely, UK) at a 1/25 dilution, for 15 minutes before application of the primary antibody. Sections were then incubated for 30 minutes at room temperature with the anti-CD8 mAb DK25 (DAKO) diluted 1/100; then biotinylated rabbit anti-mouse IgG and avidin-biotin-peroxidase complex (both DAKO). The final incubation was with the chromogen 3,3′-diaminobenzidine tetrahyrdochloride. Toluidine blue was used as the counterstain.

### Hypoxic culture

$2 \times 10^6$ THP-1 cells (purchased from the American Type Culture Collection, Rockville, MD) were cultured in 2 ml serum-free RPMI 1640 supplemented with 0.1 % BSA and 50 μM β-mercaptoethanol, in each well of a 6-well plate. The cells were then incubated for up to 24 h at 37˚C under normoxia (5% $CO_2$, in air) or hypoxia (gassed with 5% $CO_2$, balanced $N_2$ until < 0.1% $O_2$, unpublished data) in a modular incubation chamber.

### Northern analysis

Total RNA (15 μg) was run on a 1% agarose-formaldehyde gel as described in (Turner et al, 1999). Densitometric analysis was carried out using NIH Image 1.61.

### Cell counting and statistics

The CCR1 expressing cells in 15 High Power Fields (HPF) were compared with the number of $CD8^+$ cells in 15 HPF in the serial section. 15 HPF corresponded to a total tumour area of 1.095 $mm^2$,

 © 2001 Cancer Research Campaign so the counts were expressed as cells $mm^{-2}$. As the data were not normally distributed, the nonparametric Spearman's rank correlation was used to calculate *P* values (Altman, 1991).

### RESULTS

### Chemokine receptor mRNA expression in ovarian epithelial tumours

25 biopsies from human epithelial ovarian cancer were analysed for chemokine receptor mRNA expression by RPA. RPA was performed with a template set containing probes for CCR1, 2, 2a, 2b, 3, 4, 5, 8, and the 'housekeeping' genes *GAPDH* and *L32*. All the CC chemokine receptor mRNAs were expressed by a control PBMC preparation (Figure 1A). In contrast, CC chemokine receptor mRNA was weakly expressed in the solid tumour biopsies. CCR1 was the only CC chemokine receptor present in the majority of the samples, with 75% of the biopsies positive for this receptor mRNA. Less than 15% of the biopsies were positive for the remaining CC chemokine receptor mRNAs (Figure 1A and B).

Leukocytes are likely to be the main source of chemokine receptor mRNA in the tumours and their mRNA would have been diluted by other cells in the tumour microenvironment. To confirm the RPA results, therefore, the more sensitive technique of RT-PCR was used on RNA from the same biopsies. No mRNA was detected for CCR2, 2a, 2b, 3 or 5 in samples that were negative by RPA. CCR1 was detected in those samples positive by RPA (Figure 1C). 72% of the samples also gave a positive signal for CCR4 although these were negative by RPA (Figure 1C).

### Characterisation of chemokine receptor mRNA expressing cells in ovarian tumour biopsies

We used in situ hybridisation to mRNA on frozen sections from 11 biopsies to localise CCR1 and CCR4 expression. 9 of the 11 biopsies were positive for CCR1 by RPA and CCR1 could be detected on cells in these biopsies by ISH. Numbers of CCR1 expressing cells ranged from 13.7-83.1 cells $mm^{-2}$, with a median 41.4 cells $mm^{-2}$. 2 of the biopsies used for ISH were negative by RPA, yet very occasional cells (mean 2.7 cells $mm^{-2}$) could be found that were positive for CCR1 mRNA, demonstrating the increased sensitivity of ISH compared with RPA. CCR1 mRNA localised mainly to clusters of cells in the stromal areas of the ovarian tumour biopsies and the distribution was consistent with expression by infiltrating cells (Figure 2). Epithelial tumour cells did not appear to express CCR1 mRNA. As described above, CCR4 mRNA was detected by RT-PCR but not by RPA in RNA from solid tumours. As might be expected, individual cells expressing CCR4 were rare (Figure 2). They were seen in 4/11 biopsies, with only 2-3 positive cells detected in the entire section. This demonstrates the extreme sensitivity of RT-PCR, but suggests that RPA and ISH give more meaningful data on chemokine receptor mRNA expression in tissue samples. Thus a combination of techniques for measuring RNA suggests that expression of CCR 2, 3, 5 and 8 in the solid tumour microenvironment is extremely low both in terms of RNA and number of expressing cells, although cells that might be expected to express these receptors are present.

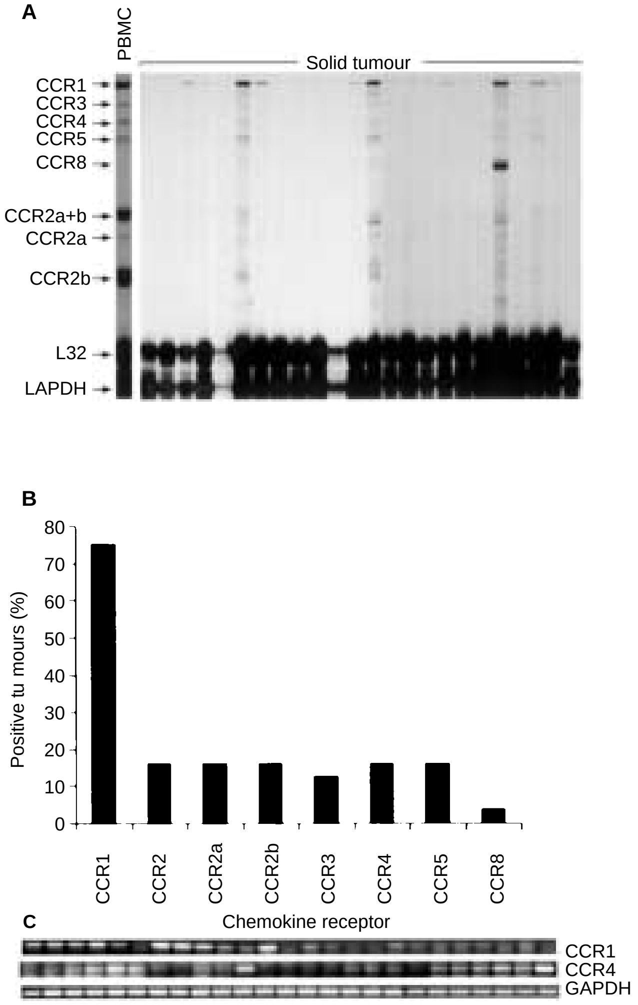

**Figure 1** (**A**) RNase protection assay of CC chemokine receptor expression in normal PBMC and solid human ovarian tumour biopsies. (**B**) Percentage of samples expressing CC chemokine receptor mRNA in solid human ovarian tumours, using the data derived from the RPA shown in Figure 1 A. Only CCR1 is expressed by the majority of solid tumours. (**C**) The presence of CCR1 and CCR4 mRNA in the solid tumours was confirmed by RT-PCR. Amplification of GAPDH is shown as a loading control

### Correlation of CCR1 expression in solid tumours with CD8 lymphocytes

In a previous publication, we characterised the infiltrating leukocytes in ovarian cancer as $CD3^+$/$CD8^+$/$CD45RO^+$ lymphocytes and $CD68^+$ macrophages (Negus et al, 1997). In this study, we found that many of these lymphocytes express CCR1. Immunohistochemistry for CD8 and ISH to CCR1 mRNA was performed on sequential sections. CCR1 expression was often seen at the same location as $CD8^+$ T cells in the sequential section (Figure 3). The number of cells expressing CCR1 was counted in 15 HPF, corresponding to a total tumour area of 1.095 $mm^2$. Similarly, the number of cells expressing CD8 was counted in 15 HPF in the sequential section; only those cells with obvious nuclei and good cytoplasmic staining were scored. A possible correlation was found between the number of cells expressing CCR1 and the number of infiltrating $CD8^+$ T-cells in individual

*© 2001 Cancer Research Campaign* 

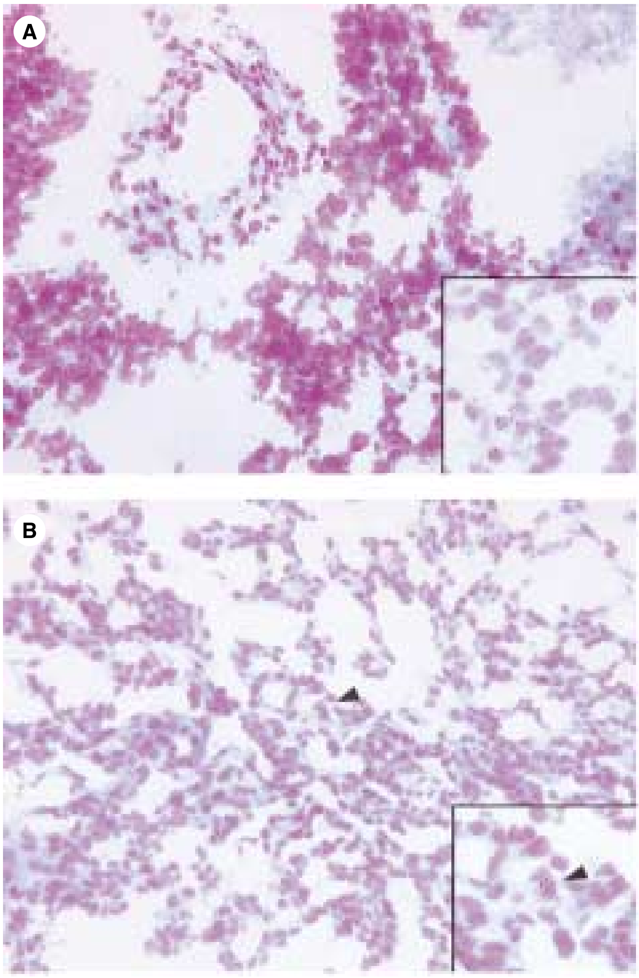

**Figure 2** Localisation of CCR1 and CCR4 mRNA by in situ hybridisation. The figure shows a cluster of cells expressing CCR1, ×400 (**A**); a cluster of cells expressing CCR1, ×1000 (inset); a single cell expressing CCR4, ×400 (**B**) and the same cell ×1000 (inset)

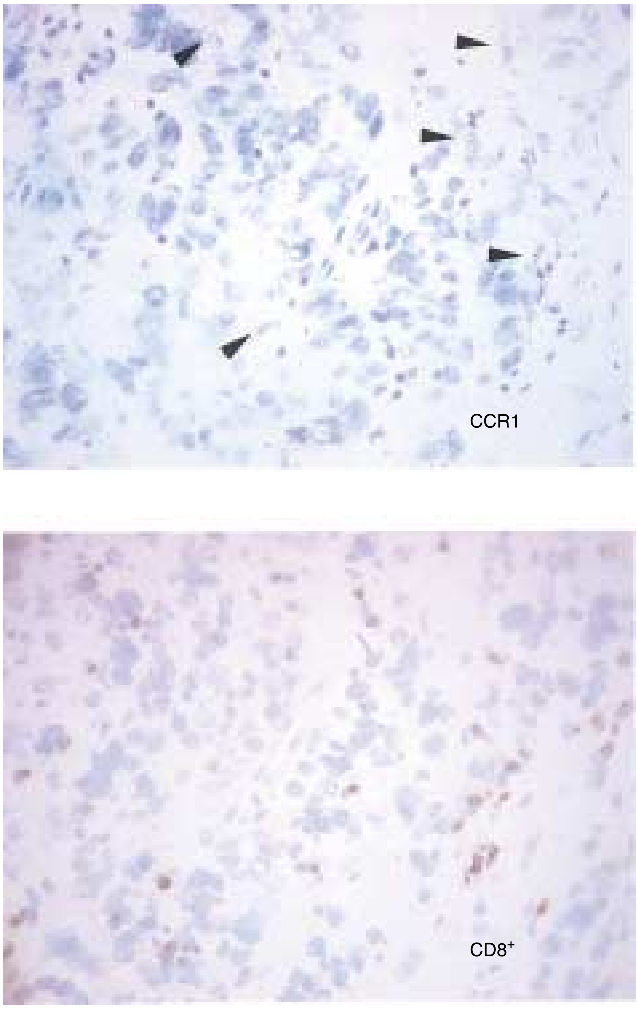

**Figure 3** Co-localisation of CCR1 expression and CD8 expression in the same field of view in serial sections (×400). Arrows indicate cells expressing CCR1

tumour sections ($r_s$ = 0.682; *P* = 0.031). A proportion of the cells expressing CCR1 also had the morphological characteristics of macrophages. Immunohistochemistry to CD68 was not of sufficient quality to obtain statistical correlation with the ISH results but there were examples of concordance between CD68 positivity and CCR1 expression on the sequential sections (data not shown).

### A range of CC chemokine mRNA is detected in ovarian tumours

We used RT-PCR to screen for 6 of the CC chemokines (CCL2, CCL3, CCL4, CCL5, CCL8 and CCL22) that bind to the receptors studied above, in the same biopsy samples. CCL2, CCL3, CCL4, CCL5 and CCL8, were detected in more than 80% of the solid tumour samples, while CCL22 was detected in 6/25 samples (Figure 4). This agrees with previous work from our laboratory where CCL2, 3, 4 and 5 were detected by ISH (Negus et al, 1997). Thus, a range of chemokine mRNA are expressed, despite the variability in mRNA expression of chemokine receptors, suggesting that their action may be controlled at the level of the receptor.

### Regulation of CCR1 expression in the tumour microenvironment

A number of agents regulate CCR1 such as LPS (Sica et al, 1997), and the cytokines IL-12, IFN-γ and IFN-α (Bonecchi et al, 1999; Colantonio et al, 1999; Zella et al, 1999). However, these cytokines have not been reported in ovarian tumours (Burke et al, 1996). TNF-α is a predominant cytokine in the ovarian tumour microenvironment (Naylor et al, 1993) and previous reports suggested that the presence of this pro-inflammatory cytokine might down-regulate the CCL2 receptor, CCR2b (Sica et al, 2000). To study the in vitro effect of this cytokine on CCR1, we used the monocytic cell line THP-1 which expresses both CCR1 and CCR2b mRNA. Treatment of THP-1 cells with 1 and 10 ng $ml^{-1}$ TNF-α did not influence CCR1 mRNA expression (data not shown); 100 ng $ml^{-1}$ was toxic to the cells. Another environmental factor likely to influence cell behaviour is intratumoural oxygen tension. We therefore studied the influence of hypoxia on CCR1 and CCR2b expression.

### The regulation of CC chemokine receptors by hypoxia

Regions of hypoxia are common in solid tumours due to the chaotic and intermittent blood supply, and the high metabolic rate

 *© 2001 Cancer Research Campaign*

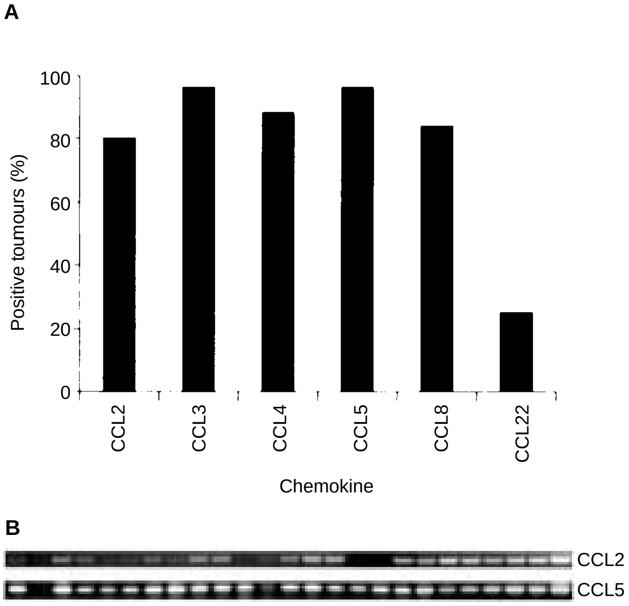

**Figure 4** The mRNA expression of 6 CC chemokines was assessed by RT-PCR in 25 solid human ovarian tumour biopsies. (**A**) The percentage of samples positive for each chemokine is shown. (**B**) Representative gels for CCL2 and CCL5 RT-PCR

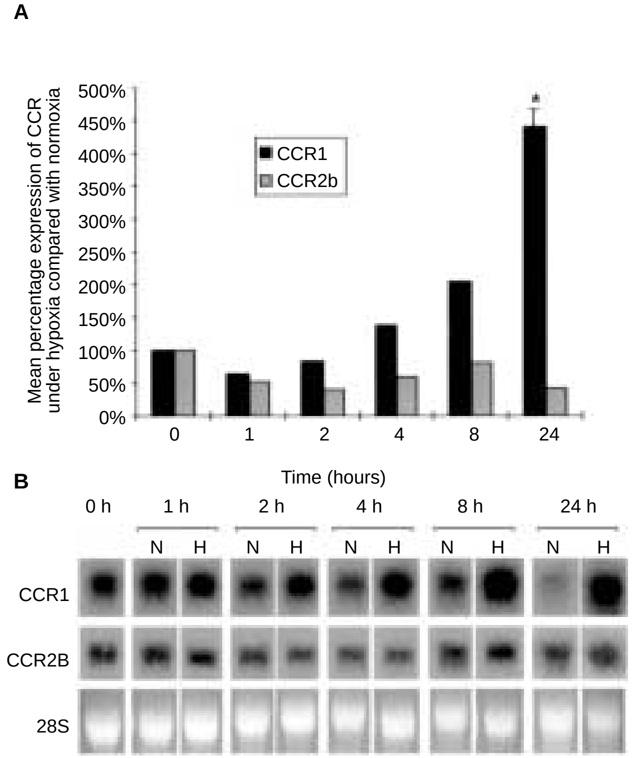

**Figure 5** Northern analysis of CCR1 and CCR2b mRNA expression in THP-1 cells. THP-1 cells were cultured under hypoxic conditions for 24 h. (**A**) The expression under hypoxia is shown as a percentage of the expression under normoxia, normalised for the housekeeping gene b-actin. *24 h timepoint includes the SEM (n = 6); all other timepoints were representative of 2 experiments. (**B**) Representative Northern blots for CCR1 and CCR2b showing mRNA expression at each timepoint, with the 28S band from the ethidium bromide stained gel shown as a loading control. N = normoxia; H = hypoxia of tumour cells (Vaupel et al, 1998). Macrophages accumulate in regions of necrosis, which are usually hypoxic (Negus et al, 1998) and hypoxia can affect the migration of THP-1 cells and monocytes, but not lymphocytes (Turner et al, 1999). Thus THP-1 cells were cultured under hypoxic conditions for 24 h, and CCR1/2b mRNA expression was assayed by Northern blot analysis during this time (Figure 5). After 24 h hypoxia, the expression of CCR1 mRNA (normalised for the housekeeping gene, β-actin) was approximately 5-fold higher compared with cells cultured under normoxia. During the same time, CCR2b mRNA expression was relatively constant (Figure 5).

### DISCUSSION

The tumour microenvironment of human epithelial ovarian cancer comprises a mixture of normal and tumour cells (Burke et al, 1996; Negus et al, 1997). There is considerable variability within and between individual biopsies. However, a median of 37% of the area of each tissue section is occupied by stromal cells, 43% by epithelial tumour areas, the rest being regions of necrosis and space (real or artefactual) (Negus et al, 1997). Within the stromal areas, the majority of infiltrating leukocytes are $CD3^+/CD8^+/CD45RO^+$ T cells and $CD68^+$ macrophages. These cells are also found amongst the epithelial tumour cells.

The cytokine context of the ovarian tumour microenvironment is generally proinflammatory. There is frequent expression of TNF-α, IL-1α, IL-6 and several growth factors, but little expression of IFN-γ, IL-2, IL-4 and IL-7 that are required for lymphocyte functions (Burke et al, 1996). CC chemokines are also present and they may control the leukocyte infiltrate (Negus et al, 1997). The CC chemokine CCL2 is expressed by both tumour cells and infiltrating cells (Negus et al, 1995). Correlation between the number of CCL2 expressing cells and the $CD8^+$ and $CD68^+$ infiltrate suggested that CCL2 was a predominant chemokine but it was not clear how the chemokine network was functioning.

In contrast to our findings with CC chemokines, receptor expression is limited in solid tumours, with CCR1 predominating. CCR1 mRNA expression correlated with the $CD8^+$ infiltrate, and CCR1 mRNA also appeared to be expressed by macrophages. CCL2 is not a ligand for this receptor and the major receptor for CCL2, CCR2b, was not detected in the solid tumours, as has previously been reported (Sica et al, 2000). CCR2 may be down-regulated by proinflammatory cytokines present in solid tumours (Sica et al, 1997). We did not find any expression of CC chemokine receptors on ovarian tumour cells, or ovarian cancer cell lines.

The present study demonstrates the importance of studying both chemokines and their receptors in a tissue and leads us to suggest that the CC chemokine network in solid tumours of ovarian cancer is controlled at the level of CC chemokine receptor. We suggest that CC chemokines such as CCL2 attract peripheral leukocytes into the tumour tissue but once there, they lose the ability to respond to this and other CC chemokines because of receptor down-regulation. This traps cells in the tumour microenvironment and changes their chemokine response profile.

Control of the chemokine network by receptor expression makes sense. As a range of chemokines are expressed by the tumours there will be conflicting chemoattractant gradients that an individual cell can follow. These gradients may be difficult to regulate with chemokines being retained by the extracellular matrix. Chemokine receptor expression, in contrast, can be rapidly modulated by LPS, chemokines, cytokines (Sica et al, 1997;

© 2001 Cancer Research Campaign 

Mantovani et al, 1998; Signoret et al, 1998; Bonecchi et al, 1999; Colantonio et al, 1999; Zella et al, 1999) and, as shown in this paper, hypoxia. Thus, microenvironmental control of chemokine receptor expression will track an individual cell along an appropriate gradient.

Hypoxia is an important factor in solid tumours; it can be an important prognostic indicator in gynaecological cancer (Hockel et al, 1998) and has been shown to regulate the expression of the chemokines CCL2 (Negus et al, 1998) and CXCL8 (IL-8) (Xu et al, 1999) and the chemokine receptor CXCR1 (Grutkoski et al, 1999). Moreover, it is being exploited as a therapeutic strategy in tumours (Griffiths et al, 2000). Hypoxia was a strong stimulus for the upregulation of CCR1. The promoter region of this chemokine receptor has been cloned by Lee et al (direct submission to Genbank, accession no. AF051305). Analysis of the promoter sequence using MatInspector V2.2 (Quandt et al, 1995) revealed 2 potential binding sites (at –892 and –760 from the transcriptional start site) for the transcription factor HIF-1. This transcription factor is stabilised under hypoxia, and controls the expression of a number of target genes including VEGF and erythropoietin (Forsythe et al, 1996; Maxwell and Ratcliffe, 1998) through a hypoxia responsive element (HRE). HREs always contain the sequence RCGTG (where R is a purine) which is critical for HIF-1 binding; the flanking residues are also important, but no strong consensus has been observed. The presence of potential HIF-1-binding sites suggests that CCR1 could be a target for transcriptional regulation by HIF-1, and could account for the upregulation seen in THP-1 cells under hypoxic conditions. However, further work is required to determine if any of these binding sites are functional. Preliminary analysis of the CCR2 promoter sequences showed no similar HRE consensus sequence in the promoter region.

Hypoxia may have time-dependent effects on cell migration because we have found that hypoxia is a rapid and potent ‘stop’ signal, inhibiting migration after as little as 30 minutes of exposure (Grimshaw and Balkwill, 2001; Negus et al, 1998; Turner et al, 1999), and this may account for the accumulation of macrophages seen in areas of necrosis in solid ovarian tumours. The upregulation of chemokine receptor expression reported here peaked much later at 24 hours.

Tumour-associated macrophages may contribute to tumour growth and spread providing growth and survival cytokines, angiogenic factors and proteases for remodelling the extracellular matrix (Mantovani, 1994; Mantovani et al, 1992). Inhibiting the tumour infiltrate may inhibit growth and spread of the malignant cells. Chemokine receptors are an obvious target for such intervention. A recent study of acute and chronic graft rejection models is of interest. Graft survival in mice with a targeted gene disruption of CCR1 was significantly prolonged and permanent engraftment occurred in some of these mice (Gao et al, 2000). We propose that the CCR1 receptor may also be a therapeutic target in human epithelial ovarian cancer.

### ACKNOWLEDGEMENTS

We would like to thank Frances Burke and Matt Grimshaw from our laboratory for help with in situ hybridisation and analysis of the CCR1 promoter; and George Elia and other members of the ICRF histopathology lab.

### REFERENCES

Altman DG (1991) *Practical statistics for medical research*. Chapman and Hall: London

Baggiolini M and Moser B (1997) Blocking chemokine receptors. *J Exp Med* **186**: 1189-1191

Bonecchi R, Polentarutti N, Luini W, Borsatti A, Bernasconi S, Locati M, Power C, Proudfoot A, Wells TNC, Mackay C, Mantovani A and Sozzani S (1999) Up-regulation of CCR1 and CCR3 and induction of chemotaxis to CC chemokines by IFN-gamma in human neutrophils. *J Immunol* **162**: 474-479

Bottazzi B, Ghezzi P, Taraboletti G, Salmona M, Colombo N, Bonazzi C, Mangioni C and Mantovani A (1985) Tumor-derived chemotactic factor(s) from human ovarian carcinoma: evidence for a role in the regulation of macrophage content of neoplastic tissues. *Int J Cancer* **36**: 167-173

Burke F, Relf M, Negus R and Balkwill F (1996) A cytokine profile of normal and malignant ovary. *Cytokine* **8**: 578-585

Car BD, Meloni F, Luisetti M, Semenzato G, Gialdroni-Grassi G and Walz A (1994) Elevated IL-8 and MCP-1 in the bronchoalveolar lavage fluid of patients with idiopathic pulmonary fibrosis and pulmonary sarcoidosis. *Am J Respir Crit Care Med* **149**: 655-659

Clore GM and Gronenborn AM (1995) Three-dimensional structures of alpha and beta chemokines. *Faseb J* **9**: 57-62

Colantonio L, Iellem A, Clissi B, Pardi R, Rogge L, Sinigaglia F and Dambrosio D (1999) Upregulation of integrin alpha 6/beta 1 and chemokine receptor CCR1 by interleukin-12 promotes the migration of human type 1 helper T cells. *Blood* **94**: 2981-2989

Ebnet K and Vestweber D (1999) Molecular mechanisms that control leukocyte extravasation: the selectins and the chemokines. *Histochem Cell Biol* **112**: 1-23

Forsythe JA, Jiang BH, Iyer NV, Agani F, Leung SW, Koos RD and Semenza GL (1996) Activation of vascular endothelial growth factor gene transcription by hypoxia-inducible factor 1. *Mol-Cell-Biol* **16**: 4604-4613

Gao JL, Wynn TA, Chang Y, Lee EJ, Broxmeyer HE, Cooper S, Tiffany HL, Westphal H, Kwon-Chung J and Murphy PM (1997) Impaired host defense, hematopoiesis, granulomatous inflammation and type 1-type 2 cytokine balance in mice lacking CC chemokine receptor 1. *J Exp Med* **185**: 1959-1968

Gao W, Topham PS, King JA, Smiley ST, Csizmadia V, Lu B, Gerard CJ and Hancock WW (2000) Targeting of the chemokine receptor CCR1 suppresses development of acute and chronic cardiac allograft rejection. *J Clin Invest* **105**: 35-44

Griffiths L, Binley K, Iqball S, Kan O, Maxwell P, Ratcliffe P, Lewis C, Harris A, Kingsman S and Naylor S (2000) The macrophage-a novel system to deliver gene therapy to pathological hypoxia. *Gene Ther* **7**: 255-262

Grimshaw MJ and Balkwill FR (2001) Inhibition of monocyte and macrophage chemotaxis by hypoxia and inflammation-a potential mechanism. *Eur J Immunol* **31**: 480-489

Grutkoski PS, Graeber CT, Damico R, Keeping H and Simms HH (1999) Regulation of IL-8RA (CXCR1) expression in polymorphonuclear leukocytes by hypoxia/reoxygenation. *J Leukoc Biol* **65**: 171-178

Hockel M, Schlenger K, Hockel S, Aral B, Schaffer U and Vaupel P (1998) Tumor hypoxia in pelvic recurrences of cervical cancer. *Int J Cancer* **79**: 365-369

Hosaka S, Akahoshi T, Wada C and Kondo H (1994) Expression of the chemokine superfamily in rheumatoid arthritis. *Clin Exp Immunol* **97**: 451-457

Howard OMZ, Oppenheim JJ and Wang JM (1999) Chemokines as molecular targets for therapeutic intervention. *J Clin Immunol* **19**: 280-292

Kurihara T, Warr G, Loy J and Bravo R (1997) Defects in macrophage recruitment and host defense in mice lacking the CCR2 chemokine receptor. *J Exp Med* **186**: 1757-1762

Lu B, Rutledge BJ, Gu L Fiorillo J, Lukacs NW, Kunkel SL, North R, Gerard C and Rollins BJ (1998) Abnormalities in monocyte recruitment and cytokine expression in monocyte chemoattractant protein 1-deficient mice. *J Exp Med* **187**: 601-608

Mantovani A (1994) Tumor-associated macrophages in neoplastic progression: a paradigm for the in vivo function of chemokines. *Lab Invest* **71**: 5-16

Mantovani A, Bottazzi B, Colotta F, Sozzani S and Ruco L (1992) The origin and function of tumor-associated macrophages. *Immunol Today*, **13**: 265-270

Mantovani A, Allavena P, Vecchi A and Sozzani S (1998) Chemokines and chemokine receptors during activation and deactivation of monocytes and dendritic cells and in amplification of th1 versus th2 responses. *Int J Clin Lab Res* **28**: 77-82

Mattei S, Colombo MP, Melani C, Silvani A, Parmiani G and Herlyn M (1994) Expression of cytokine/growth factors and their receptors in human melanoma and melanocytes. *Int J Cancer* **56**: 853-857

*© 2001 Cancer Research Campaign*

Maxwell P and Ratcliffe P (1998) Regulation of expression of the erythropoietin gene. *Curr Opin Hematol* **5**: 166-170

Naylor MS, Stamp GW and Balkwill FR (1990) Investigation of cytokine gene expression in human colorectal cancer. *Cancer Res* **50**: 4436-4440

Naylor MS, Stamp GW, Foulkes WD, Eccles D and Balkwill FR (1993) Tumor necrosis factor and its receptors in human ovarian cancer. Potential role in disease progression. *J Clin Invest* **91**: 2194-2206

Negus RP, Stamp GW, Relf MG, Burke F, Malik ST, Bernasconi S, Allavena P, Sozzani S, Mantovani A and Balkwill FR (1995) The detection and localization of monocyte chemoattractant protein-1 (MCP-1) in human ovarian cancer. *Journal of Clinical Investigation* **95**: 2391-2396

Negus RP, Stamp GW, Hadley J and Balkwill FR (1997) Quantitative assessment of the leukocyte infiltrate in ovarian cancer and its relationship to the expression of C-C chemokines. *Am J Pathol* **150**: 1723-1734

Negus RP, Turner L, Burke F and Balkwill FR (1998) Hypoxia down-regulates MCP-1 expression: implications for macrophage distribution in tumors. *J Leukoc Biol* **63**: 758-765

Quandt K, Frech K, Karas H, Wingender E and Werner T (1995) Matlnd and MatInspector: new fast and versatile tools for detection of consensus matches in nucleotide sequence data. *Nucleic Acids Res* **23**: 4878-4884

Schweickart VL, Epp A, Raport CJ and Gray PW (2000) CCR11 is a functional receptor for the monocyte chemoattractant protein family of chemokines. *J Biol Chem* **275**: 9550-9556

Sica A, Saccani A, Borsatti A, Power CA, Wells TN, Luini W, Polentarutti N, Sozzani S and Mantovani A (1997) Bacterial lipopolysaccharide rapidly inhibits expression of C-C chemokine receptors in human monocytes. *Journal of Experimental Medicine*, **185**: 969-974

Sica A, Saccani A, Bottazzi B, Bernasconi S, Allavena P, Gaetano B, Fei F, LaRosa G, Scotton C, Balkwill F and Mantovani A (2000) Defective Expression of the Monocyte Chemotactic Protein-1 Receptor CCR2 in Macrophages Associated with Human Ovarian Carcinoma. *J Immunol* **164**: 733-738

Signoret N, Rosenkilde MM, Klasse PJ, Schwartz TW, Malim MH, Hoxie JA and Marsh M (1998) Differential regulation of cxcr4 and ccr5 endocytosis. *J Cell Sci* **111**: 2819-2830

Takeya M, Yoshimura T, Leonard EJ and Takahashi K (1993) Detection of monocyte chemoattractant protein-1 in human atherosclerotic lesions by an anti-monocyte chemoattractant protein-1 monoclonal antibody. *Hum Pathol* **24**: 534-539

Turner L, Scotton C, Negus R and Balkwill F (1999) Hypoxia inhibits macrophage migration. *Eur J Immunol* **29**: 2280-2287

Turner SJ, Domin J, Waterfield MD, Ward SG and Westwick J (1998) The CC chemokine monocyte chemotactic peptide-1 activates both the class I p85/p110 phosphatidylinositol 3-kinase and the class II P13K-C2alpha. *J Biol Chem* **273**: 25987-25995

Van Coillie E, Froyen G, Nomiyama H, Miura R, Fiten P, Van Aelst I, Van Damme J and Opdenakker G (1997) Human monocyte chemotactic protein-2: cDNA cloning and regulated expression of mRNA in mesenchymal cells. *Biochem Biophys Res Commun* **231**: 726-730

Vaupel P, Thews O, Kelleher DK and Hoeckel M (1998) Current status of knowledge and critical issues in tumor oxygenation. Results from 25 years research in tumor pathophysiology. *Adv Exp Med Biol* **454**: 591-602

Wang JM, Deng XY, Gong WH and Su SB (1998) Chemokines and their role in tumor-growth and metastasis. *J Immunol Methods* **220**: 1-17

Xu L, Xie K, Mukaida N, Matsushima K and Fidler IJ (1999) Hypoxia-induced elevation in interleukin-8 expression by human ovarian carcinoma cells. *Cancer Res* **59**: 5822-5829

Yoong KF, Afford SC, Jones R, Aujla P, Qin S, Price K, Hubscher SG and Adams DH (1999) Expression and function of CXC and CC chemokines in human malignant liver tumors: a role for human monokine induced by gamma-interferon in lymphocyte recruitment to hepatocellular carcinoma. *Hepatology* **30**: 100-111

Zella D, Barabitskaja O, Casareto L, Romerio F, Secchiero P, Reitz MS, Gallo RC and Weichold FF (1999) Recombinant IFN-alpha (2b) increases the expression of apoptosis receptor CD95 and chemokine receptors CCR1 and CCR3 in monocytoid cells. *J Immunol* **163**: 3169-3175

Zlotnik A and Yoshie O (2000) Chemokines: A new classification system and their role in immunity. *Immunity* **12**: 121-127

*© 2001 Cancer Research Campaign*
